# Supplementary material for: Predation and fragmentation portrayed in the statistical structure of prey time series
Source: BMC Ecol. 2009 May 6;9:10. doi: 10.1186/1472-6785-9-10 (PMC2689204; doi:10.1186/1472-6785-9-10)
Supplement: Additional file 2 — Voles and related classes ODDox Documentation. ODDox documentation of the agent-based model (ALMaSS) applied by Hendrichsen et al. The documentation is started by activating main.html. [file 1472-6785-9-10-S2.zip › Vole_ODDox/globals_0x6d.html]

ALMaSS ODDox: Class Members

- Main Page
- Related Pages
- Classes
- Files

- File List
- File Members

- All
- Functions
- Variables
- Typedefs
- Enumerations
- Enumerator
- Defines

- \_
- a
- b
- c
- d
- f
- g
- h
- i
- l
- m
- n
- o
- p
- r
- s
- t
- u
- v
- w

Here is a list of all file members with links to the files they belong to:

### - m -

- MaleMovement
  : Vole\_all.cpp- MaleReproductFinish
    : Vole\_all.cpp- MaleTerritoryRange
      : Vole\_all.cpp- MaleTerritoryRangeSlope
        : Vole\_all.cpp- MaturationChance
          : Vole\_all.cpp- MaxFemaleTerrSize
            : Vole\_all.cpp- MaxMaleTerrSize
              : Vole\_all.cpp- MaxStarvationDays
                : Vole\_all.cpp- MaxWeightF
                  : Vole\_all.cpp- MaxWeightM
                    : Vole\_all.cpp- MHabQualThreshold1
                      : Vole\_all.cpp- MHabQualThreshold2
                        : Vole\_all.cpp- MHabQualThreshold3
                          : Vole\_all.cpp- MinFemaleMovement
                            : Vole\_all.cpp- MinFVoleHabQual
                              : Vole\_all.cpp- MinMaleMovement
                                : Vole\_all.cpp- MinMVoleHabQual
                                  : Vole\_all.cpp- MinReproAgeF
                                    : Vole\_all.cpp- MinReproAgeM
                                      : Vole\_all.cpp- MinReproWeightF
                                        : Vole\_all.cpp- MinReproWeightM
                                          : Vole\_all.cpp- molluscicide
                                            : treatment.h- MoveToLessFavourable
                                              : VolePopulationManager.cpp
                                              , Vole\_all.cpp- mow
                                                : treatment.h- MutationChance
                                                  : GeneticMaterial.cpp

---

Generated on Thu Jan 22 14:13:48 2009 for ALMaSS ODDox by 
 1.5.6
